# Evolutionary Relationships of Unclassified Coronaviruses in Canadian Bat Species

**DOI:** 10.3390/v16121878

**Published:** 2024-12-04

**Authors:** Ayo Yila Simon, Maulik D. Badmalia, Sarah-Jo Paquette, Jessica Manalaysay, Dominic Czekay, Bishnu Sharma Kandel, Asma Sultana, Oliver Lung, George Giorgi Babuadze, Nariman Shahhosseini

**Affiliations:** 1 Centre for Vector-Borne Diseases, National Centre for Animal Diseases, Canadian Food Inspection Agency, Lethbridge, AB T1J 3Z4, Canada; ayo.simon@gov.ab.ca (A.Y.S.); maulik.badmalia@gmail.com (M.D.B.); sarahjo.paquette@gmail.com (S.-J.P.); jessica.manalaysay@inspection.gc.ca (J.M.); dominic.czekay@gmail.com (D.C.); bishnu.kandel@inspection.gc.ca (B.S.K.); 2 Departments of Chemistry & Biochemistry, University of Lethbridge, Lethbridge, AB T1K 3M4, Canada; 3 National Centre for Foreign Animal Diseases, Canadian Food Inspection Agency, Winnipeg, MB R3E 3M4, Canada; asma.sultana@inspection.gc.ca (A.S.); oliver.lung@inspection.gc.ca (O.L.); 4 Department of Biological Sciences, University of Manitoba, Winnipeg, MB R3T 2N2, Canada; 5 Department of Microbiology & Immunology, University of Texas Medical Branch, Galveston, TX 77555, USA; gebabuad@utmb.edu; 6 Department of Biological Sciences, University of Lethbridge, Lethbridge, AB T1K 3M4, Canada

**Keywords:** Alphacoronavirus, Eptacovirus, *Eptesicus fuscus*, *Myotis lucifugus*, *Myotis californicus*, emerging zoonoses, phylogeny, Canada

## Abstract

Bats are recognized as natural reservoirs for an array of diverse viruses, particularly coronaviruses, which have been linked to major human diseases like SARS-CoV and MERS-CoV. These viruses are believed to have originated in bats, highlighting their role in virus ecology and evolution. Our study focuses on the molecular characterization of bat-derived coronaviruses (CoVs) in Canada. Tissue samples from 500 bat specimens collected in Canada were analyzed using pan-coronavirus RT-PCR assays to detect the presence of CoVs from four genera: Alpha-CoVs, Beta-CoV, Gamma-CoV, and Delta-CoV. Phylogenetic analysis was performed targeting the RNA-dependent RNA polymerase (RdRP) gene. Our results showed an overall 1.4% CoV positivity rate in our bat sample size. Phylogenetic analysis based on the ~600 bp sequences led to the identification of an unclassified subgenus of Alpha-CoV, provisionally named Eptacovirus. The findings contribute to a better understanding of the diversity and evolution of CoVs found in the bat species of Canada. The current study underscores the significance of bats in the epidemiology of CoVs and enhances the knowledge of their genetic diversity and potential impact on global public health.

## 1. Introduction

Coronaviruses (CoVs) have been linked to severe illnesses in human populations as a result of spillover events [1]. In 2003 in China, the emergence of severe acute respiratory syndrome (SARS) was thought to have originated from bats and was subsequently amplified by civets [2]. Similarly, in 2012 in Saudi Arabia, the emergence of Middle East respiratory syndrome (MERS) was found to have originated from bats and then amplified in dromedary camels [3,4]. Since then, CoVs have gained global attention due to their profound impact on public health, economies, and societies. The emergence of SARS-CoV-2 in 2019, which was thought to have spread from bats or pangolins to humans as a result of a spillover event [5], once again stunned the public health sector globally and highlighted the pressing need to elucidate this enigmatic virus family.

CoVs belong to the subfamily *Orthocoronavirinae* within the family *Coronaviridae* of the order *Nidovirales*. Subfamily *Orthocoronavirinae* comprises four distinct genera, namely *Alphacoronavirus* (Alpha-CoV), *Betacoronavirus* (Beta-CoV), *Gammacoronavirus* (Gamma-CoV), and *Deltacoronavirus* (Delta-CoV) [6,7]. This classification is defined based on both the evolutionary relationships and genomic structures of CoVs, as proposed by the International Committee on the Taxonomy of Viruses (ICTV). Alpha-CoVs and Beta-CoVs are known to exclusively infect mammals, while Gamma-CoVs and Delta-CoVs primarily infect birds but have the potential to infect mammals [7,8,9].

CoVs are the largest group of RNA viruses, with genome sizes varying from 25 to 32 kb and virions spanning 118 to 140 nm in diameter. The structural foundation of CoVs lies in their enveloped nature with a single-stranded (ss) positive-sense (+) RNA genome. The viral genome comprises five primary open reading frames (ORFs) encoding the structural proteins, namely the replicase polyproteins (ORF1a and ORF1ab), spike (S), envelope (E), and membrane (M) glycoproteins, along with the nucleocapsid protein (N) occurring in the 5′ to 3′ order [10].

Currently, a total of seven endemic human CoVs (HCoV) have been identified as pathogens causing respiratory tract infections in humans. This includes the Alpha-CoVs HCoV-229E and HCoV-NL63, as well as the Beta-CoVs HCoV-OC43, HCoV-HKU1, MERS-CoV, SARS-CoV, and SARS-CoV-2 [11,12]. Among them, the four human-infecting CoVs, namely HCoV-229E, HCoV-NL63, HCoV-OC43, and HCoV-HKU1, typically induce mild respiratory symptoms, whilst the three others, namely MERS-CoV, SARS-CoV, and SARS-CoV-2, are recognized as more pathogenic, causing deadly outbreaks, which underscores the critical importance of continuous global CoV surveillance [13,14].

It is believed that all known HCoVs have emerged from mammalian sources [11]. HCoV-OC43 and HKU1 are thought to have likely emerged from rodents, while other human CoVs have genetic similarity to bat CoVs, suggesting potential direct or indirect spillover events from bats to humans [11,15]. In addition to the public health importance of CoVs, the Alpha-CoVs and Beta-CoVs can also significantly impact livestock, causing a substantial disease burden and negatively impacting the agricultural industry. Porcine transmissible gastroenteritis virus (TGEV), porcine enteric diarrhea virus (PEDV), bovine coronavirus (BCoV), and the recently identified swine acute diarrhea syndrome CoV (SADS-CoV) are examples of CoVs with veterinary medicine importance [16,17,18].

The 21st century has witnessed the emergence of three novel CoVs with epidemic or pandemic potential—SARS-CoV (2002–2003), MERS-CoV (2012), and SARS-CoV-2 (2019–present). Despite the emergence of these viruses and the discovery of other animal CoVs related to SARS-CoV-2, the natural origins of many of emerging CoVs have remained elusive. Lessons from these outbreaks have underscored the need for more in-depth research to decipher the natural evolutionary origins of these viruses. Bats are natural reservoirs of several viruses that are thought to cause deadly diseases in humans, including rabies. The emergence of these viruses in humans appears to be caused by direct spillover events or through intermediate hosts [19].

Despite numerous studies investigating the presence of CoVs and other viruses in bats across the globe, particularly Europe and Asia, there is still limited information about the diversity of bat CoVs in North America. Given that Canada and the USA share the largest international land border in the world, it is expected that they share common bat species and viruses. In fact, several bat CoVs have been identified in bats in the USA, but little is known about Canada. Both the Canadian Food Inspection Agency (CFIA) in Lethbridge, Alberta and the CFIA Fallowfield Laboratory in Ottawa, Ontario conduct rabies testing in animals for Western and Eastern Canada [20,21], respectively, but do not test for CoVs. To this end, we performed the molecular screening of CoVs in bat specimens that were negative for rabies, which were obtained from samples submitted from different Canadian provinces for rabies diagnosis.

## 2. Materials and Methods

### 2.1. Ethics Statement

The current study used post-mortem bat specimens that were previously submitted to the CFIA Lethbridge Laboratory located in Lethbridge, for rabies diagnosis. Therefore, specific ethics approval was not required for this investigation (refer to the Institutional Review Board Statement).

### 2.2. Sampling

**Figure 1:**
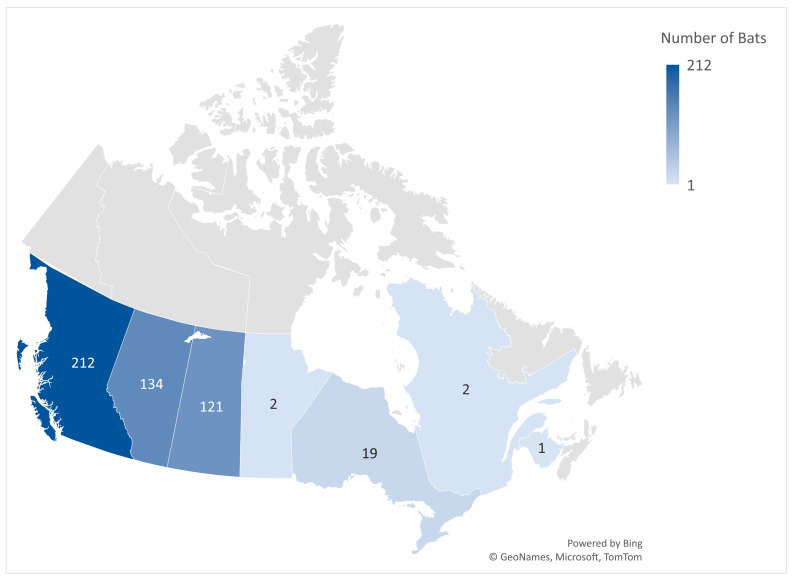
The number of bat specimens per province included in this study.

All 500 bat specimens included in this study were collected through a passive surveillance process from 8 January 2020 to 13 December 2022, from seven Canadian provinces, including British Columbia (n = 212), Alberta (n = 134), Saskatchewan (n = 121), Manitoba (n = 2), Ontario (n = 19), Quebec (n = 2), and New Brunswick (n = 1). All specimens were previously submitted to the CFIA in Lethbridge for rabies diagnostic testing (Figure 1). The collection site information for nine bat specimens was not submitted to the CFIA.

**Figure 2:**
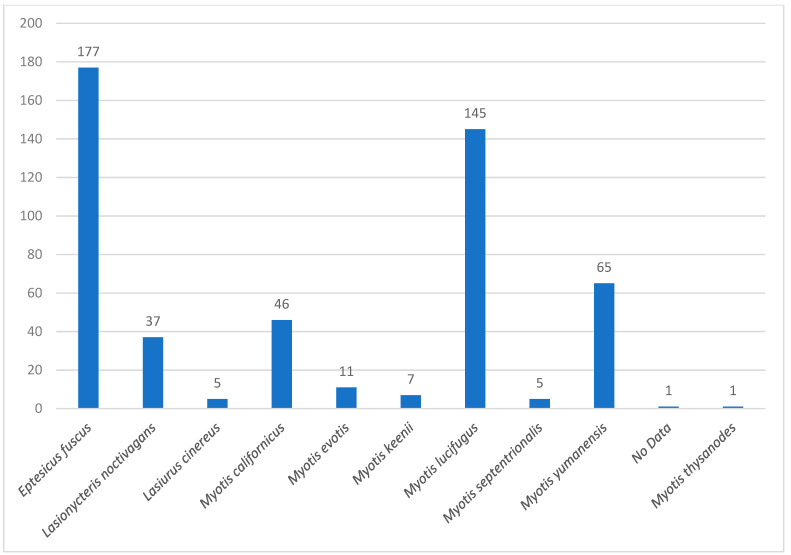
The prevalence of different bat species included in this study.

Bat species were identified using a morphological characteristic key for Canadian bat species “https://batwatch.ca/bat-species-central-canada (accessed on 27 November 2024)”. Bat specimens that tested negative for rabies were frozen at −20 °C until further analysis. The collected bat species included in this study were *Eptesicus fuscus* (big brown bat), *Lasionycteris noctivagans* (silver-haired bat), *Lasiurus cinereus* (hoary bat), *Myotis californicus* (California bat), *Myotis evotis* (long-eared bat), *Myotis keenii* (Keen’s long-eared bat), *Myotis lucifugus* (little brown bat), *Myotis septentrionalis* (northern long-eared bat), *Myotis yumanensis* (Yuma bat), and *Myotis thysanodes* (fringe bat) (Figure 2).

### 2.3. Bat Tissue Harvesting and RNA Extraction

The biosafety guidelines of the National Center for Animal Disease Laboratory, CFIA, Lethbridge were followed for the processing of the rabies-negative bat specimens in an advanced Containment Level 2 facility. Before dissection, each bat carcass was decontaminated using a 70% ethanol solution and then air-dried on absorbent paper for approximately 10 min. Post-dissection, available tissue such as the brain (when present), heart, intestines, kidneys, liver, lungs, and spleen was extracted from each specimen. Subsequently, all harvested organs were pooled and divided into two equal portions for each bat. One set of the pooled organs was stored at −80 °C in a 2 mL tube containing RNAlater (Thermo Fisher Scientific in Waltham, MA, USA) for future analysis.

The other set was kept in a 3 mL tube containing sterile 5 mm stainless-steel beads and Trizol^®^ reagent (Thermo Fisher Scientific in Waltham, MA, USA) for RNA extraction. The bat tissue was homogenized using oscillations at 1800 per minute using a Qiagen TissueLyser II^®^ (Qiagen, Hilden, Germany). Following homogenization, the samples were centrifuged at 15,000 rpm for 15 min. The resulting supernatant was transferred to a new tube and used for RNA isolation.

The homogenized tissue samples (in Trizol) were further processed using the RNeasy Maxi Kit (Qiagen, Toronto, ON, Canada), following the manufacturer’s protocols, with slight modifications to extract the RNA. Briefly, 500 µL of the supernatant from the homogenized tissue in TRIzol^TM^ reagent (Invitrogen^TM^) was combined with 300 µL of chloroform (Sigma-Aldrich, MilliporeSigma, Mississauga, ON, Canada). After brief vortexing, the samples were allowed to stand for 5 min at room temperature. Subsequently, the mixtures were centrifuged at 13,000× *g* at 4 °C for 10 min, resulting in the separation of the organic and aqueous layers. The aqueous layer was carefully transferred to fresh tubes, and 3 volumes of chilled absolute ethanol were added, followed by gentle pipetting to mix. This mixture was then transferred to a spin column containing RNA binding resin. The spin columns were individually washed with their respective wash buffers and then eluted using an elution buffer, following the manufacturer’s protocol. The eluted RNA was stored at −80 °C until further use.

### 2.4. Pan-Coronavirus for Screening of Bat Specimens

To screen bat specimens for CoVs from four genera, Alpha-CoV, Beta-CoV, Gamma-CoV, and Delta-CoV, RNA extracts from five bat specimens were pooled together; then, a previously designed Pan-CoV protocol based on a conserved region of the RNA-dependent RNA polymerase (RdRP) was utilized [22]. The semi-nested PCR was performed using one-step SuperScript III (Invitrogen^TM^) according to the manufacturer’s protocol. Briefly, 5 µL of RNA was mixed with 6.25 µL of 2x reaction mix, 0.3 μM of each CoV600_outF (5′-CCAARTTYTAYGGHGGWTGG-3′) and CoV600_R (5′-TGTTGWGARCARAAYTCATGWGG-3′) primer, and 0.5 µL of enzyme mix. Amplification was carried out using an Applied Biosystems™ Veriti™ Thermal Cycler (Thermo Fischer Scientific, Mississauga, Ontario, Canada). The thermal profile included a reverse transcription step at 60 °C for 1 min and extension at 50 °C for 45 min. This was followed by denaturation at 94 °C for 2 min, followed by 45 cycles of denaturation at 94 °C for 15 s, primer annealing at 53.4 °C for 30 s, and extension at 68 °C for 60 s. A final extension step occurred at 68 °C for 7 min. Subsequently, the reaction mixture was stored at 4 °C.

The DNA product generated in the first round of RT-PCR was then used as a template in a semi-nested PCR, which replaced the CoV600_outF primer used previously with CoV600_inF (5′-GGTTGGGAYTAYCCHAARTGTGA-3′), ultimately producing a 600 bp RdRp amplicon. For this step, 5 µL of DNA produced in the initial RT-PCR reaction was mixed with 12.5 µL of 2x HotStarTaq Master Mix and 0.5 μM of each primer (CoV600_inF and CoV600_R), and the final volume was adjusted to 25 µL with nuclease-free water. The amplification process included initial denaturation at 95 °C for 15 min, followed by 35 cycles of denaturation at 94 °C for 30 s, annealing at 54.5 °C for 30 s, and extension at 72 °C for 60 s. A final extension step was performed at 72 °C for 10 min.

We used a synthetic sequence that was specifically designed to be detectable by the primer sets as the positive control for PCR. The resulting PCR products were analyzed using a QIAxcel DNA Fast Analysis Kit for the QIAxcel Advanced Instrument (QIAGEN, Hilden, Germany). To determine the size of the amplicons, the QX DNA Size Marker (50–1500 bp) was run alongside the QX Alignment Marker (15–3000 bp), to identify amplicons at 600 bp size. Then, samples displaying a band at the anticipated position were purified using a PCR product clean-up kit (Zymogen Research, Irvine, CA, USA) and subsequently submitted for Sanger Sequencing (Eurofins Scientific, Louisville, KY, USA). Pools whose products were discovered through sequencing to contain a CoV were then opened, and each sample was tested separately using the aforementioned protocol.

### 2.5. Next-Generation Sequencing

The RNA extracted from big brown bat (*Eptesicus fuscus*) LET-AH-2021-RA-00661 was sent to the Canadian Food Inspection Agency National Centre for Foreign Animal Disease Genomics Unit for next-generation sequencing (NGS). Complementary DNA (cDNA) was synthesized using random hexamers and the Protoscript II First Strand cDNA synthesis kit (New England Biolabs). The NGS library was prepared following the Total Nucleic Acids Library Preparation EF Kit 2.0 for Viral Pathogen Detection and Characterization and the comprehensive viral research probe panel using the Twist target enrichment Standard Hybridization v1 Protocol, as recommended by the manufacturer (Twist Bioscience, San Francisco, CA, USA). The enriched DNA library was sequenced on an Illumina NextSeq 1000/2000 instrument using a P1 600 cycle flow cell (Illumina, San Diego, CA, USA). Raw sequencing reads were initially analyzed for viral reads and assembled contigs using the Genome Detective Virus Tool v2.72 (genomedetective.com). Raw reads were mapped to the longest contig generated from the Genome Detective Tool in Geneious Prime (Biomatters Inc., Cambridge, MA, USA), using the Geneious iterative mapper assembler, followed by the iterative mapping of the assembled contigs to extend the genome using Geneous Prime (Biomatters Inc.). The assembled consensus sequence was aligned to the Eptesicus bat coronavirus strain 16964 from the United States (OL410609.1) in Geneious Prime, using MAFFT (v7.490) for comparison.

### 2.6. Phylogenetic Tree Construction

In addition to the sequences obtained in this study, a dataset including CoVs from diverse groups was obtained from GenBank (Supplementary Table S1). The sequences were aligned using the CLUSTAL W algorithm and a phylogenetic tree was constructed by the neighbor-joining (NJ) algorithm with sorted topologies and a 70% threshold using the Geneious Prime 2021.2.2. The bootstrap method was used to assess the branch support (1000 replicates). [23] To further analyze the precise evolutionary origins of the sequences obtained in this study, a split network was created via the EqualAngel method using the SplitsTree 4.14.8 software [24].

### 2.7. Data Availability

The DNA sequences generated by pan RT-PCR (~600 bp) were deposited at Genbank under accession numbers OR365502, OR365503, OR371599, OR371597, OR371596, OR371600, and OR371949, and the sequences generated by NGS (611 bp of RdRp gene) received an accession number of PP621596.

## 3. Results

### 3.1. Prevalence of CoVs in Bat Species

Of the 500 bat samples obtained by passive surveillance from seven Canadian provinces, seven samples from different pools (1.4%) were positive for CoV by a pan-coronavirus PCR. The positivity rates based on the number of bats obtained per province were as follows: Ontario 5.3% (1/19), Saskatchewan 2.5% (3/121), British Columbia 0.94% (2/212), and Alberta 0.75% (1/134). No bat samples from Manitoba, New Brunswick, or Quebec were positive for CoV based on the pan-CoV PCR. Among the CoV-positive bat species, the big brown bat, *Eptesicus fuscus*, had the highest percentage of CoV positivity at 71.4% (5/7), while both *Myotis californicus* (vesper bat) and *Myotis lucifugus* (little brown bat) had a 14.3% positivity rate (1/7). All other bat species analyzed were negative for CoVs (Table 1).

### 3.2. Evolutionary Relationship of Bat CoVs

**Figure 3:**
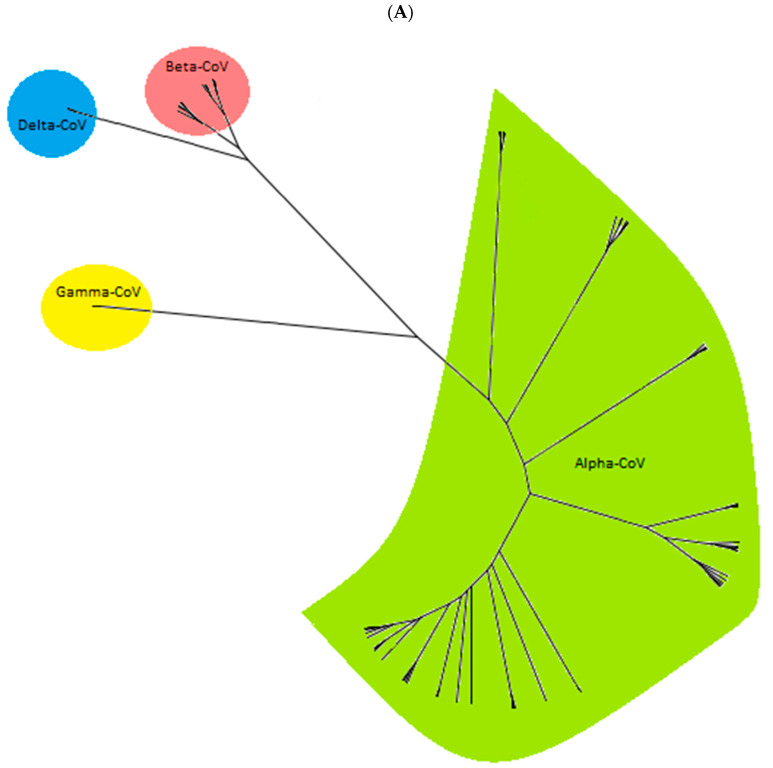

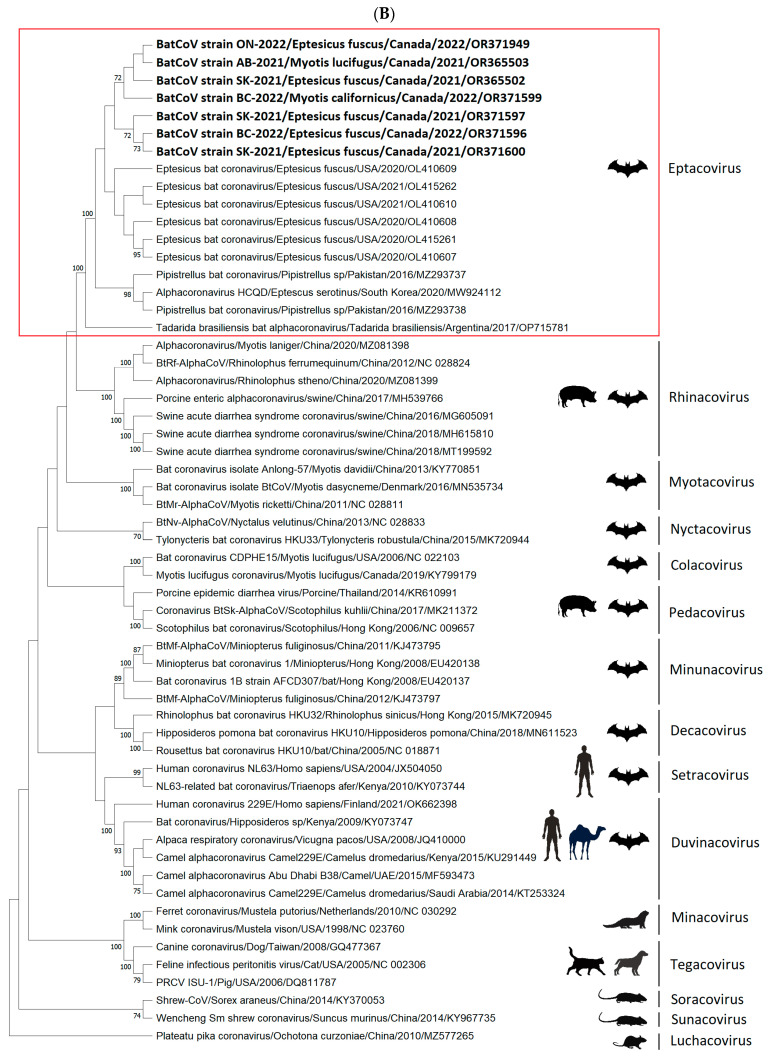

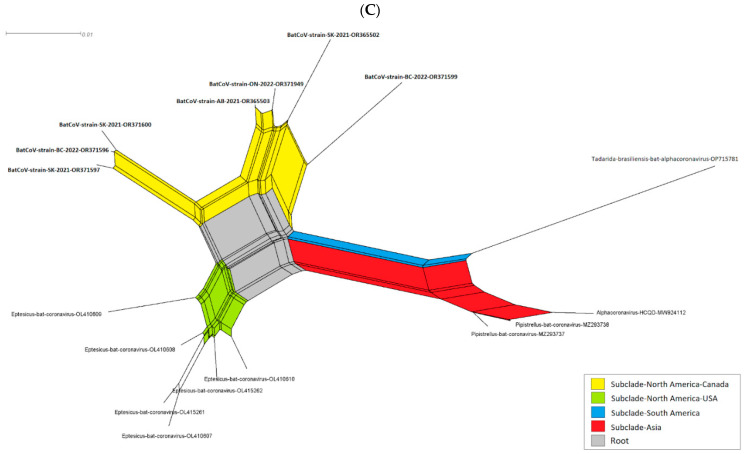
The cladogram of coronaviruses (**A**) and the phylogenetic NJ tree of AlphaCoVs were constructed from aligned sequences using tools implemented within the Geneious software 2021.2.2 (**B**). The bootstrap values and number of bootstrap replications were greater than 70% and 1000, respectively. The neighbor-net network based on the sequences of unclassified subclades (Eptacovirus) was constructed using the Split Tree software Version 4.17.1; an enlargement of Eptacovirus is shown (**C**).

The phylogenetic analysis based on the partial genome sequences of CoVs (260 bp to 547 bp) confirmed the clustering of the CoV sequences in four distinct groups, including Alpha-CoV, Beta-CoV, Delta-CoV, and Gamma-CoV (Figure 3A). The group Alpha-CoVs is further divided into 14 known CoV species, classified as Sunacovirus, Soracovirus, Minacovirus, Tegacovirus, Rhinacovirus, Luchacovirus, Myotacovirus, Decacovirus, Minunacovirus, Nyctacovirus, Setracovirus, Duvinacovirus, Pedacovirus, and Colacovirus. Interestingly, the sequences obtained from the seven positive Canadian bat samples (three species) in this study formed an unclassified subgenus with other bat CoVs from the USA, Pakistan, South Korea, and Argentina and, as a result, are provisionally named Eptacovirus (Figure 3B).

Split tree networking demonstrated that the Eptacovirus subgenus was later split into three subclades based on their geographical origins, including a South American cluster consisting of Tadarida bat CoV isolates from Argentina, an Asian cluster consisting of bat CoV isolates from Pakistan and South Korea, and the North American cluster consisting of bat CoV isolates from the USA and Canada (Figure 3C).

## 4. Discussion

In this study, we screened 500 bat specimens from Canada, spanning 10 different species, for CoVs (Figure 2), and we discovered an unclassified Alpha-CoV in seven bat specimens in Canada. Our phylogenetic analysis showed the grouping of Alpha-CoVs into 14 known species. Interestingly, all of the Alpha-CoVs sequences obtained in our study fell into an unclassified Alpha-CoV cluster, provisionally named Eptacovirus. Subsequent split tree analysis on the Eptacoviruses showed the clustering of the isolates in this subgenus based on their geographical origin, where Canadian isolates of Eptacovirus were grouped with other isolates from the USA but distinct from other Eptacovirus isolates found in South America and Asia.

In a similar study in the US, the American isolates of Eptacovirus were reported as *Eptesicus* bat CoVs (EbCoV) since they were isolated from *Eptesicus fuscus* species of bats. Schaeffer, R et al. showed that the full genome of EbCoV was found to be closely related to the bat CoV strain HKU2 and SADS-CoV, while the S gene was similar to PEDV [25]. The genetic similarity between EbCoV/Eptacovirus and swine CoVs and bat CoVs indicates the possibility of spillover events during the evolution of the virus. This is crucial because highly virulent strains of PEDV and SADS-CoV are among the most devastating pathogens that harm the swine industry worldwide. This emphasizes the need for more research to better understand the potential risk that certain viruses pose to the livestock industry. To prevent future outbreaks, researchers need to further scrutinize the zoonotic potential of bat-borne viruses (BBVs) and swine CoVs. The One Health concept can be used to systematically monitor and track the spread of novel CoVs in both humans and animal species. To prevent any spillover events, especially during and after an outbreak, the identification and characterization of viruses in wild animal reservoirs may provide invaluable knowledge that improves our understanding of virus transmission and its pathogenic capacity [26,27,28].

Over the past two decades, CoVs have garnered significant attention, especially with the emergence of the novel SARS-CoV-2 in 2019, which led to the COVID-19 pandemic, with over seven million human deaths by April 2024. COVID-19 strongly emphasized how CoVs have the potential to cause widespread health and socio-economic impacts [29]. The Alpha-CoVs hold significance in both public and animal health due to their capacity to cause diseases in humans and animals [13,30].

A crucial aspect of Alpha-CoVs is their potential for cross-species transmission, with animal reservoirs sometimes leading to the emergence of CoVs that can infect humans, underscoring the importance of consistent monitoring in animal populations to safeguard public health [31,32]. This interconnectedness between public and animal health (One Health) necessitates collaborative efforts in surveillance, vaccine development, and treatment strategies, especially considering the challenges presented by these groups of rapidly mutating viruses and the variety of species that they affect [32,33].

Bats are the source of many viral pathogens transmissible to humans and livestock, such as rabies [34]. However, one of the most prevalent viruses in bats is CoVs, the causative agent for circulating epidemics and worldwide pandemics [30,35]. The large genome size, combined with high mutation and recombination rates between homologous RNA regions, allows CoVs to adapt to new hosts and ecological niches [5,8,36]. The spillover of bat-associated viruses occurs due to a combination of factors, including an ecological opportunity for contact, virus–host molecular and cellular compatibility, and a permissive or circumvented immune response. Furthermore, climate change and human activities have resulted in close proximity between wild animals and humans, thereby increasing the chances of viral transmission to hosts [34,35]. All of these factors make it imperative for us to undertake a proactive approach to the monitoring and screening of BBVs, specifically CoVs, to prevent the future spread of zoonotic spillover diseases. Our study led to the discovery of unclassified AlphaCoVs in Canada, enhancing our understanding of CoVs with potential zoonotic transmission in the country’s bat population. Both our findings and those from previous similar studies in North America [37,38] suggest that many more bat-originated CoVs remain to be identified and described. This is of concern because CoVs are characteristically known to recombine, leading to the emergence of CoVs that are a threat to public health [39]. Taken together, our results provide more insights into the evolutionary relationships of AlphaCoVs associated with North American bats, which adds to the global epidemiology of emerging CoVs.

As the effects of climate change and socio-economic activities escalate in the upcoming years, there may be a heightened frequency of interactions between bats, humans, and domestic animals, potentially increasing the likelihood of bat virus spillover and the emergence of novel infectious diseases. Despite finding unclassified CoVs in a limited number of samples in our study, this underscores the broader issue that potentially more bat species may harbor a number of unidentified viruses with potential zoonotic capabilities. To unravel the specific roles of different bat species in this context, we advocate for an extensive study that includes a larger sample size, encompassing various virus families and including forest-dwelling bat species that have traditionally had minimal contact with humans or domestic animals. Gaining a comprehensive understanding of the viruses that bats harbor, as well as the ecological factors that facilitate their maintenance, is crucial in mitigating the risk of spillover infections and reducing the zoonotic potential.

Creating a catalogue of bat-harbored viruses, along with the molecular characterization of their genome products, would be invaluable. This would not only facilitate the early detection of a virus during the initial stages of an outbreak, epidemic, or pandemic but also aid in pinpointing reservoir host species for these viruses. Our initial study represents a modest advancement toward these ambitious research objectives.

In conclusion, these findings improve the current knowledge of the genetic diversity, classification, and evolution of the Coronavirus family. Further research is needed to determine the potential significance of this unclassified CoV as a pathogen of importance for human or animal health.

## Figures and Tables

**Table 1:** Positive bat species for unclassified Alpha-CoVs (Eptacovirus) by pan-RT-PCR.

Bat Species	Date of Collection	City/Town of Collection	Province Location	Accession Number
*Eptesicus fuscus*	04/08/2021	Saskatoon	Saskatchewan	OR365502
*Eptesicus fuscus*	13/10/2021	Moose Jaw	Saskatchewan	OR371600/PP621596 *
*Eptesicus fuscus*	08/06/2021	Regina	Saskatchewan	OR371597
*Eptesicus fuscus*	21/01/2022	Stratford	Ontario	OR371949
*Eptesicus fuscus*	26/05/2022	Summerland	British Columbia	OR371596
*Myotis lucifugus*	09/06/2021	Sylvan lake	Alberta	OR365503
*Myotis californicus*	31/05/2022	Pender Island	British Columbia	OR371599

* Note: the accession number PP621596 is for the partial RdRp sequence obtained by NGS.

## Data Availability

Data are contained within the article and Supplementary Materials.

## Supplementary Materials

The following supporting information can be downloaded at: https://www.mdpi.com/article/10.3390/v16121878/s1, Table S1. Listing the sequences retrieved from GenBank and included in the phylogenetic analysis.
