# Supplementary material for: Evolutionary Relationships of Unclassified Coronaviruses in Canadian Bat Species
Source: Viruses. 2024 Dec 4;16(12):1878. doi: 10.3390/v16121878 (PMC11680298; doi:10.3390/v16121878)
Supplement: Supplementary file 1 [file viruses-16-01878-s001.zip › viruses-3105594-supplementary.pdf]

Table S1. Listing the sequences retrieved from GenBank and included in the phylogenetic analysis.

CoV genera	Isolate	Host	Collection site-country	Collection year	GenBank accession number
Alphacoronavirus	Eptesicus bat coronavirus	Eptesicus fuscus	USA	2020	OL415261
	Eptesicus bat coronavirus	Eptesicus fuscus	USA	2021	OL415262
	Eptesicus bat coronavirus	Eptesicus fuscus	USA	2021	OL410610
	Eptesicus bat coronavirus	Eptesicus fuscus	USA	2020	OL410608
	Eptesicus bat coronavirus	Eptesicus fuscus	USA	2020	OL410607
	Eptesicus bat coronavirus	Eptesicus fuscus	USA	2020	OL410609
	Alphacoronavirus HCQD	Eptescus serotinus	South Korea	2020	MW924112
	Pipistrellus bat coronavirus	Pipistrellus sp	Pakistan	2016	MZ293737
	Pipistrellus bat coronavirus	Pipistrellus sp	Pakistan	2016	MZ293738
	Alphacoronavirus	Myotis laniger	China	2020	MZ081398
	Alphacoronavirus	Rhinolophus stheno	China	2020	MZ081399
	Bat coronavirus 1B strain AFCD307	bat	Hong Kong	2008	EU420137
	Bat coronavirus CDPHE15	Myotis lucifugus	USA	2006	NC 022103
	Bat coronavirus isolate Anlong-57	Myotis davidii	China	2013	KY770851
	Bat coronavirus isolate BtCoV	Myotis dasycneme	Denmark	2016	MN535734
	Bat coronavirus	Hipposideros sp	Kenya	2009	KY073747
	BtMf-AlphaCoV	Miniopterus fuliginosus	China	2011	KJ473795
	BtMf-AlphaCoV	Miniopterus fuliginosus	China	2012	KJ473797
	Alpaca respiratory coronavirus	Vicugna pacos	USA	2008	JQ410000
	BtMr-AlphaCoV	Myotis ricketti	China	2011	NC 028811
	BtNv-AlphaCoV	Nyctalus velutinus	China	2013	NC 028833
	BtRf-AlphaCoV	Rhinolophus ferrumequinum	China	2012	NC 028824
	Camel alphacoronavirus Abu Dhabi B38	Camel	UAE	2015	MF593473
	Camel alphacoronavirus Camel229E	Camelus dromedarius	Kenya	2015	KU291449
	Camel alphacoronavirus Camel229E	Camelus dromedarius	Saudi Arabia	2014	KT253324
	Canine coronavirus/	Dog	Taiwan	2008	GQ477367
	Coronavirus BtSk-AlphaCoV	Scotophilus kuhlii	China	2017	MK211372
	Feline infectious peritonitis virus	Cat	USA	2005	NC 002306
	Ferret coronavirus	Mustela putorius	Netherlands	2010	NC 030292
	Hipposideros pomona bat coronavirus HKU10	Hipposideros pomona	China	2018	MN611523
	Human coronavirus 229E	Homo sapiens	Finland	2021	OK662398
	Human coronavirus NL63	Homo sapiens	USA	2004	JX504050
	Miniopterus bat coronavirus 1	Miniopterus	Hong Kong	2008	EU420138
	Mink coronavirus	Mustela vison	USA	1998	NC 023760

	Myotis lucifugus coronavirus	Myotis lucifugus	Canada	2019	KY799179
	NL63-related bat coronavirus	Triaenops afer	Kenya	2010	KY073744
	Plateatu pika coronavirus	Ochotona curzoniae	China	2010	MZ577265
	Porcine enteric alphacoronavirus	swine	China	2017	MH539766
	Porcine epidemic diarrhea virus	Porcine	Thailand	2014	KR610991
	PRCV ISU-1	Pig	USA	2006	DQ811787
	Rhinolophus bat coronavirus HKU32	Rhinolophus sinicus	Hong Kong	2015	MK720945
	Rousettus bat coronavirus HKU10	bat	China	2005	NC 018871
	Scotophilus bat coronavirus	Scotophilus	Hong Kong	2006	NC 009657
	Shrew-CoV	Sorex araneus	China	2014	KY370053
	Swine acute diarrhea syndrome coronavirus	swine	China	2016	MG605091
	Swine acute diarrhea syndrome coronavirus	swine	China	2018	MH615810
	Swine acute diarrhea syndrome coronavirus	swine	China	2018	MT199592
	Tadarida brasiliensis bat alphacoronavirus	Tadarida brasiliensis	Argentina	2017	OP715781
	Tylonycteris bat coronavirus HKU33	Tylonycteris robustula	China	2015	MK720944
	Wencheng Sm shrew coronavirus	Suncus murinus	China	2014	KY967735
Betacoronavirus	Bat coronavirus RaTG13	Rhinolophus affinis	China	2013	MN996532
	SARS-CoV-2	Homo sapiens	China	2019	NC_045512
	Bat SARS coronavirus HKU3-1	bat	China	2005	DQ022305
	SARS-CoV	Homo sapiens	Canada	2003	NC_004718
	Hypsugo bat coronavirus HKU25	Hypsugo pulveratus	China	2014	KX442565
	Human coronavirus HKU1	Homo sapiens	France	2005	HM034837
	Rousettus bat coronavirus HKU9	bat	China	2006	NC_009021
	Pipistrellus bat coronavirus HKU5	bat	China	2006	NC_009020
	Rousettus bat coronavirus GCCDC1	Eonycteris spelaea	Singapore	2016	MT350598
	Tylonycteris bat coronavirus HKU4	bat	China	2006	NC_009019
	Bat Hp-betacoronavirus	Hipposideros pratti	China	2013	NC_025217
Gammacoronavirus	Avian coronavirus isolate Beaudette-FU	Gallus gallus	Germany	2021	MW847253
	Turkey coronavirus	Turkey	Canada	2007	NC_010800
Deltacoronavirus	Porcine coronavirus HKU15	Pig	USA	2014	KJ462462
	Sparrow deltacoronavirus	Sparrow	USA	2017	MG812377
